# Characterization of D-Allulose-3-Epimerase From *Ruminiclostridium papyrosolvens* and Immobilization Within Metal-Organic Frameworks

**DOI:** 10.3389/fbioe.2022.869536

**Published:** 2022-04-14

**Authors:** Jiaming Yang, Dexun Fan, Fengguang Zhao, Ying Lin, Suiping Zheng, Shuangyan Han

**Affiliations:** ^1^ Guangdong Key Laboratory of Fermentation and Enzyme Engineering, School of Biology and Biological Engineering, South China University of Technology, Guangzhou, China; ^2^ School of Light Industry and Engineering, South China University of Technology, Guangzhou, China

**Keywords:** allulose, metal-organic frameworks, enzyme immobilization, ruminiclostridium papyrosolven, d-allulose 3-epimerase

## Abstract

D-allulose is one sort of C-3 epimer of D-fructose with the low calorie (0.4 kcal/g) and high sweetness (70% of the relative sweetness of sucrose), which can be biosynthesized by D-allulose-3-epimerase (DAE). In this work, we report the characterization of a novel DAE from *Ruminiclostridium papyrosolvens* (RpDAE) by genome mining approach. The activity of RpDAE reached maximum at pH 7.5 and 60°C, supplemented with 1 mM Co^2+^. Using D-fructose (500 g/L) as the substrate for epimerization reaction, RpDAE produced D-allulose (149.5 g/L). In addition, RpDAE was immobilized within the microporous zeolite imidazolate framework, ZIF67, by *in situ* encapsulation at room temperature. The synthesized bio-composites were characterized by powder X-ray diffraction and Fourier transform infrared spectroscopy. RpDAE-ZIF67 maintained 56% of residual activity after five reaction cycles. This study provides helpful guidance for further engineering applications and industrial production of D-allulose.

## Introduction

Vascular risk factors, exemplified by type 2 diabetes, hypertension, and obesity, have become health concerns worldwide. The quantity of type 2 diabetes victims in 2017 is assessed to be 415 million, and 31.1% of adults (13.9 billion) have hypertension (Wyss et al., 2020). In addition, NCD Risk Factor Collaboration predicts that the global obesity incidence rate will reach 18% for men and 21% for women by 2025 under existing trends (Trends in Adult body-mass, 2016). Amassing proof demonstrates that excessive caloric intake contributes to the development of these chronic diseases (Xia et al., 2021) (Johnson et al., 2007). Thusly, developing and intaking low-calorie food supplements can be the practical methodology to defuse vascular risks (Chung et al., 2012). D-allulose (D-*ribo*-2-hexylose, C_6_H_12_O_6_), initially named D-psicose, is one sort of rare sugar. Because of its low bioavailability, D-allulose keeps up with 70% of the relative sweetness of sucrose (Chung et al., 2012) with energy of only 0.4 kcal/g. U.S. Food and Drug Administration has ratified D-allulose as “generally recognized as safe” since 2012 (GRN No. 400), which makes it an attractive sugar substitute. The presence of D-allulose in nature is very scant, which is found in a small number of plants exemplified by wheat (Miller and Swain, 1960) and *Itea* (Ayers et al., 2014) and a few processed foods, such as commercial fructose syrup mixtures and steamed treated coffee (Oshima et al., 2006).

The scarcity of D-allulose in nature enormously limits its large-scale application. Chemical synthesis of D-allulose has many defects, including complex purification process, by-product formation, and chemical waste pollution (Chen et al., 2021). By comparison, the biosynthesis of D-allulose is more efficient, with mild reaction conditions, without by-product formation, which allows better sustainability to be achieved. All monosaccharides can be cyclically transformed by four kinds of enzymes, including polyol dehydrogenase, aldose isomerase, aldose reductase, and ketose-3-epimerase (KEase). D-allulose-3-epimerases (DAEs) are one type of KEase catalyzing reversible epimerization of D-fructose, the most abundant ketose in nature, into D-allulose (Mu et al., 2015). Promising progress has been noted in the biosynthesis of D-allulose, until now, approximately 20 DAEs from different strains have been characterized (Chen et al., 2021). Most DAEs discovered to date are of bacterial origin, mainly derived from soil bacteria, e.g., *A. tumefaciens* (Kim et al., 2006a), *C. cellulolyticum* (Mu et al., 2011), *Desmospora* sp. 8,437 (Zhang et al., 2013), and *N. thermophilus* (Jia et al., 2021). Most DAEs rely on Co^2+^ or Mn^2+^ as co-factor and are inactive in the absence of metal ions.

Although the use of enzyme is an environment-friendly strategy, the actual production of D-allulose by the enzyme faces the problems of high cost, and poor operation stability. Enzyme immobilization has been proved to be an effective way to improve robustness, ease of recovery, and continuous utilization of enzymes in industrial processes (Homaei et al., 2013). High porosity, tunable pore sizes, good thermostability, and opportune biocompatibility endow metal-organic frameworks (MOFs) with potential as matrices to immobilize biological macromolecules, such as enzymes (Liang et al., 2015). Among the various MOFs, zeolitic imidazolate frameworks (ZIFs), which are formed by self-assembly of tetrahedral metal clusters (Zn^2+^ or Co^2+^) and 2-methylimidazole ligands, have been extensively studied *in situ* encapsulation because of their mild synthetic conditions in aqueous solution (Gross et al., 2012). Lyu and colleagues (Lyu et al., 2014) pioneeringly employed this strategy with cytochrome c and ZIF8, obtaining Cyt c/ZIF8 bio-composite with 10-fold higher bioactivity over free enzyme. Rafiei and co-workers constructed lipase/ZIF67 composite and applied it in the transesterification of soybean oil to biodiesel. The biocatalytic composite maintained excellent enzymatic catalytic performance after six cycles (Rafiei et al., 2018).

In the present study, a putative DAE from *Ruminiclostridium papyrosolvens* C7 (RpDAE) was identified. The RpDAE was cloned and overexpressed as recombinant proteins in *E. coli* BL21 (DE3). The enzyme properties of purified RpDAE and its application in the biological production of D-allulose were studied. In addition, RpDAE was encapsulated by ZIF67 under mild conditions to enhance its thermal stability and reusability. The RpDAE-ZIF67 bio-composites were characterized by powder X-ray diffraction (PXRD) and Fourier transform infrared (FT-IR) spectroscopy, and the catalytic performance and reusability were also evaluated.

## Materials and Methods

### Gene Cloning, Expression, and Purification of RpDAE

The gene sequence of RpDAE (NCBI ACCESSION: WP_020816056.1) was codon-optimized for *E. coli* expression and fused with a modified His-based tag (HE tag) containing eight repeat histidine-glutamate residues (HEHEHEHEHEHEHEHE) at C-terminus, which is capable of immobilized metal ion affinity chromatography purification. The sequence was synthesized (GenScript, Nanjing, China) and subcloned into the pET-21a (+) between *Nde*I and *Xho*I restriction sites. Recombinant plasmid pET-RpDAE was cloned and transformed into *E. coli*. BL21 (DE3) for protein expression.

Recombinant strains were inoculated into 10 ml of Luria-Bertani (LB) medium. When needed, ampicillin was added into LB medium at the concentration of 100 μg/ml. Then, strains were cultured at 37°C with shaking at 200 rpm overnight. The seed was transferred into 100 ml of LB medium and after cultivated at 37°C with shaking at 200 rpm. When cells grown to the proper optical density (OD_600_ = 0.7), 0.5 mM isopropyl-*β*-d-1-thiogalactopyranoside (IPTG) was added, and induced recombinant cells were further cultured at 15°C with shaking at 180 rpm for 16 h.

Recombinant cells were harvested by centrifugation at 8,000*g* for 5 min at 4°C. Subsequently, cells were washed thrice in lysis buffer (50 mM Tris-HCl, 100 mM NaCl, pH 7.5). Then, cells were lysed by sonication at 30 amplitudes (pulse on for 3 s and pulse off for 3 s) for 30 min over the ice. The cell debris was removed by centrifugation at 10,000*g* for 5 min at 4°C, and the supernatant was obtained for further purification. Enzyme with HE tag was trapped on His Trap™ FF column (Cytiva, MA, USA) at a flow rate of 0.5 ml/min. Wash buffer (50 mM Tris-HCl, 10 mM imidazole, 0.5 M NaCl, pH 7.5) was used to elute unbound proteins, and the target enzyme was eluted by elution buffer (50 mM Tris-HCl, 300 mM imidazole, 0.5 M NaCl, pH 7.5). Eluant was further dialyzed to remove imidazole with 50 mM Tris-HCl (pH 7.5) and concentrated by Amicon^®^ Ultra filter (10 kDa) (Merck, USA). The protein concentration was measured by Bradford Assay (Thermo Fisher, MA, USA). The purified protein was loaded onto sodium dodecyl sulfate–polyacrylamide gel electrophoresis (SDS-PAGE) gel for the determination of molecular mass and purity.

### Enzyme Assay

The enzyme activity was determined by quantitative determination of product converted from the substrate. Data for this study was collected by high-performance liquid chromatography (HPLC) system, linked to a 2424 evaporative light scattering detector and an xBridge BEH amide column (all from Waters, MA, USA). The temperatures of the detector and column were set at 65°C and 35°C, respectively. The mobile phase was acetonitrile and water mixture (80:20, v/v, with 0.1% v/v ammonia added) at a flow rate of 1 ml/min. The reaction system incorporated D-fructose (50 g/L), 1 mM Co^2+^, appropriate amount of RpDAE or RpDAE-ZIF67, and 50 mM KH_2_PO_4_/Na_2_HPO_4_ (pH 7.5). The reaction was performed at 60°C for 5 min and terminated by boiling for 5 min. One unit (U) of RpDAE activity was defined as the amount of enzyme required to catalyze 1 μmol of D-allulose within the unit time (min) under the reaction condition.

### Effect of Metal Ions and Substrate Specificity

To determine the influence of metal ions on the RpDAE activity, the activity was examined under standard enzyme assay except for supplemented with different metal ions (Mg^2+^, Cu^2+^, Co^2+^, Ni^2+^, Mn^2+^, Ca^2+^, and Zn^2+^) at the final concentration of 1 mM. The activity measured without metal ions was defined as 100% relative activity. The substrate specificity of RpDAE was tested by adding different ketoses (D-allulose, D-fructose, D-sorbose, and D-tagatose) into the reaction system as substrates. The reaction conditions were standard.

### Bioconversion of D-Allulose

To determine the bioconversion from D-fructose to D-allulose, 2 μM purified RpDAE was added with 1 mM Co^2+^ and D-fructose (500 g/L) in 50 mM KH_2_PO_4_/Na_2_HPO_4_ buffer (pH 7.5) at 50°C. Samples were taken at designated time intervals and diluted tenfold. The yields of the accumulative D-allulose were detected by HPLC.

### Effect of Temperature and pH

To study the effect of temperature on RpDAE activity, RpDAE was added in KH_2_PO_4_/Na_2_HPO_4_ buffer (pH 7.5) at temperatures varying from 40°C to 80°C. To investigate the effect of the pH on the activity of RpDAE, the reaction was conducted at 60°C across a pH range of 6–10 in MES buffer (50mM, pH 6.0) or KH_2_PO_4_/Na_2_HPO_4_ buffer (50 mM, pH 7.0–10.0).

### Preparation and Characterization of RpDAE-ZIF67

RpDAE-ZIF67 was synthesized by *in situ* approach in the aqueous solution. Experimentally, 2 ml of purified RpDAE (2 mg/ml) and 2 ml of cobalt nitrate hexahydrate (0.04 M) were mixed with 2-methylimidazole (1.2 M, 2 ml) in distilled water and stirred for 1 h at ambient temperature. The sample solution was aged for 7 h and collected by centrifugation at 6,000 rpm for 20 min. Subsequently, samples were washed three times with distilled water. It was freeze-dried for 12 h. PXRD data were collected by SmartLab 9 kW X-ray diffractometer (Rigaku, Tokyo, Japan) with Cu-K*α* radiation at 2θ from 5° to 40°. FT-IR measured was performed by Nicolet iS20 spectroscopy (Thermo Scientific, MA, USA) in the range of 400–2,500 cm^−1^.

## Results and Discussion

### Sequence Analysis of RpDAE

The genome mining approach has turned out to be a promising way toward the detection of novel industrial enzymes, such as lipase (Vorapreeda et al., 2015), *ß*-glucosidase (Zou et al., 2012), and laccase (Fang et al., 2011). To explore novel DAE applicable to biosynthesis D-allulose, the amino acid sequence of *Clostridium cellulolyticum* DAE (GenBank: ACL75304.1) with significant thermostability was chosen as the template to BLAST in the NCBI database. The sequence that encoded a deduced DAE (NCBI: WP_020816056.1) from *Ruminiclostridium papyrosolvens* C7 with 59% amino acid identity with *C. cellulolyticum* DAE was selected. *R. papyrosolvens* strain was initially isolated from mud in Massachusetts, and its whole-genome shotgun sequence data was uploaded to NCBI database in 2013 (Zepeda et al., 2013), with the NCBI accession number PRJNA201398.

**FIGURE 1:**
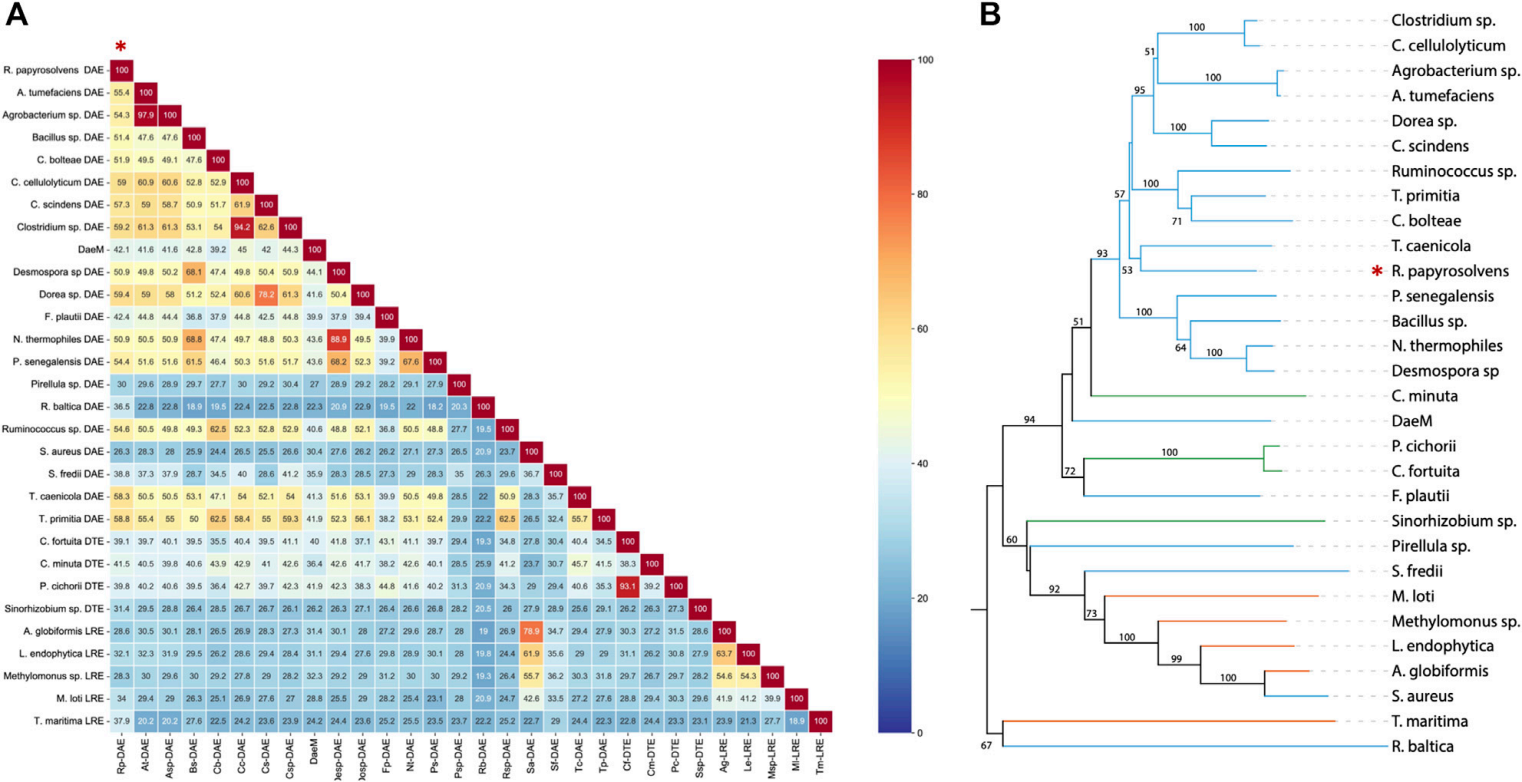
**(A)** Sequence homology analysis of reported KEases. **(B)** Phylogenetic relationships of reported KEases. Blue treetops on behalf of DAE; green treetops on behalf of DTE; orange treetops on behalf of LRE.

As presented in comparison of amino acid sequence (Figure 1A), deduced DAE from *R. Papyrosolvens* showed maximum homology identity with DAE from *Dorea* sp. (59.4%, GenBank: CDD07088.1), followed by DAE from *Clostridium* sp. (59.2%, GenBank: EDP19602.1). Except for DAEs from *Pirellula* sp., *R. baltica*, *S. aureus*, and *S. fredii* origin, there was more than 40% of homology identity between RpDAE and other characterized DAEs, while existed relatively low (less than 40%) homology identity with most D-tagatose-3-epimerases and l-ribulose-3-epimerases. Phylogenetic analysis with previously characterized KEases revealed its evolutionary relationship with DAE from genera of *T. caenicola* (Figure 1B).

**FIGURE 2:**
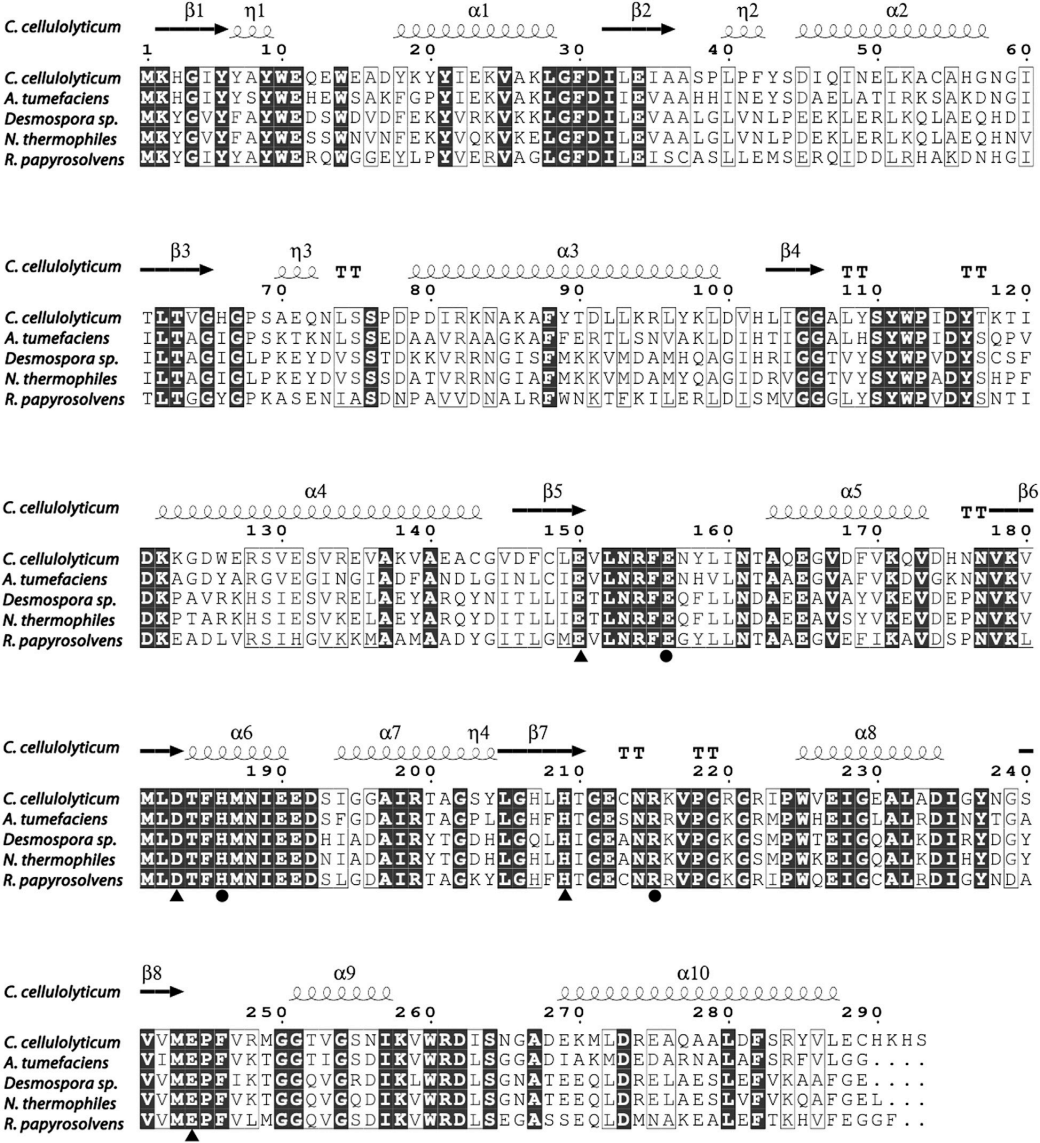
Structure-based sequence alignment between *R. papyrosolvens* DAE and mentioned DAEs. The secondary structural elements of *C. cellulolyticum* (PDB: 3VNI) are presented. Identical and similar amino acid residues are marked in black and frame, respectively. Triangle mark as metal ions binding site, and circle mark as substrate (D-fructose) O-1, O-2, and O-3 binding sites.

So far, the crystal structures of *A. tumefaciens* ATCC33970 DAE (PDB: 2HK0) (Kim et al., 2006b) and *C. cellulolyticum *H10 DAE (PDB: 3VNI) (Chan et al., 2012) have been successfully resolved, and they are homotetramers with similar monomeric structures; each subunit possesses a distinct TIM barrel structure forming by eight β-sheets and α-helices, and the catalytic activity center is strictly conserved (Jia et al., 2021). Multiple amino acid sequences alignment was performed between characterized DAEs and RpDAE (Figure 2). Crucial residues of RpDAE for the catalytic activity, including metal coordination sites (Glu150, Asp183, His209, and Glu244) and residues binding the O-1, O-2, O-3 of the substrate (Glu156, His186, and Arg215), were conserved with other reported DAEs. In general, these analyses indicate that RpDAE belongs to the DAE family.

### Expression and Purification of RpDAE

**FIGURE 3:**
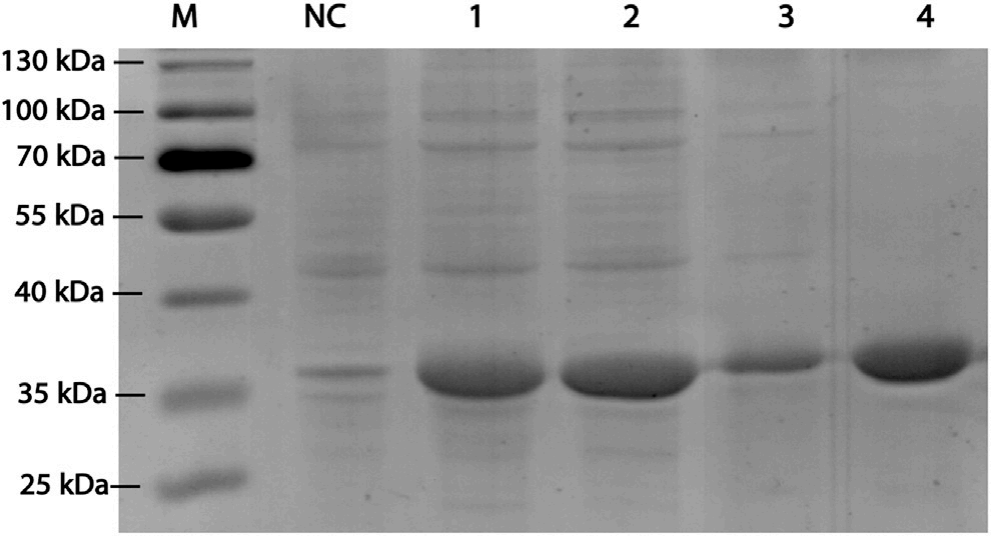
SDS-PAGE analysis for RpDAE cloned in pET-21a (+) and expressed in *E. coli* BL21 (DE3). Lane M, protein marker; Lane NC, Cell lysate without induction; Lane 1, cell lysate with induction; Lane 2, supernatant of cell lysate with induction; Lane 3, debris of cell lysate with induction; Lane 4, purified protein.

RpDAE fused with a modified histidine (HE) tag was recombinantly overexpressed in *E. coli*. BL21 (DE3) by IPTG induction. HE tag can function as promoters of both affinity (Hofström et al., 2011) and solubility (Han et al., 2018). After cell disruption, the supernatants were purified by immobilized metal affinity chromatography. SDS-PAGE visualization confirmed expression and purification of proteins of about 35 kDa (Figure 3), which was in accordance with the theoretical molecular weight of RpDAE (34.89 kDa, containing HE tag). Target proteins were mostly expressed in a soluble form under the conditions described above (Figure 3, Lane 2).

### Effect of Metal Ion and Substrate Specificity

**FIGURE 4:**
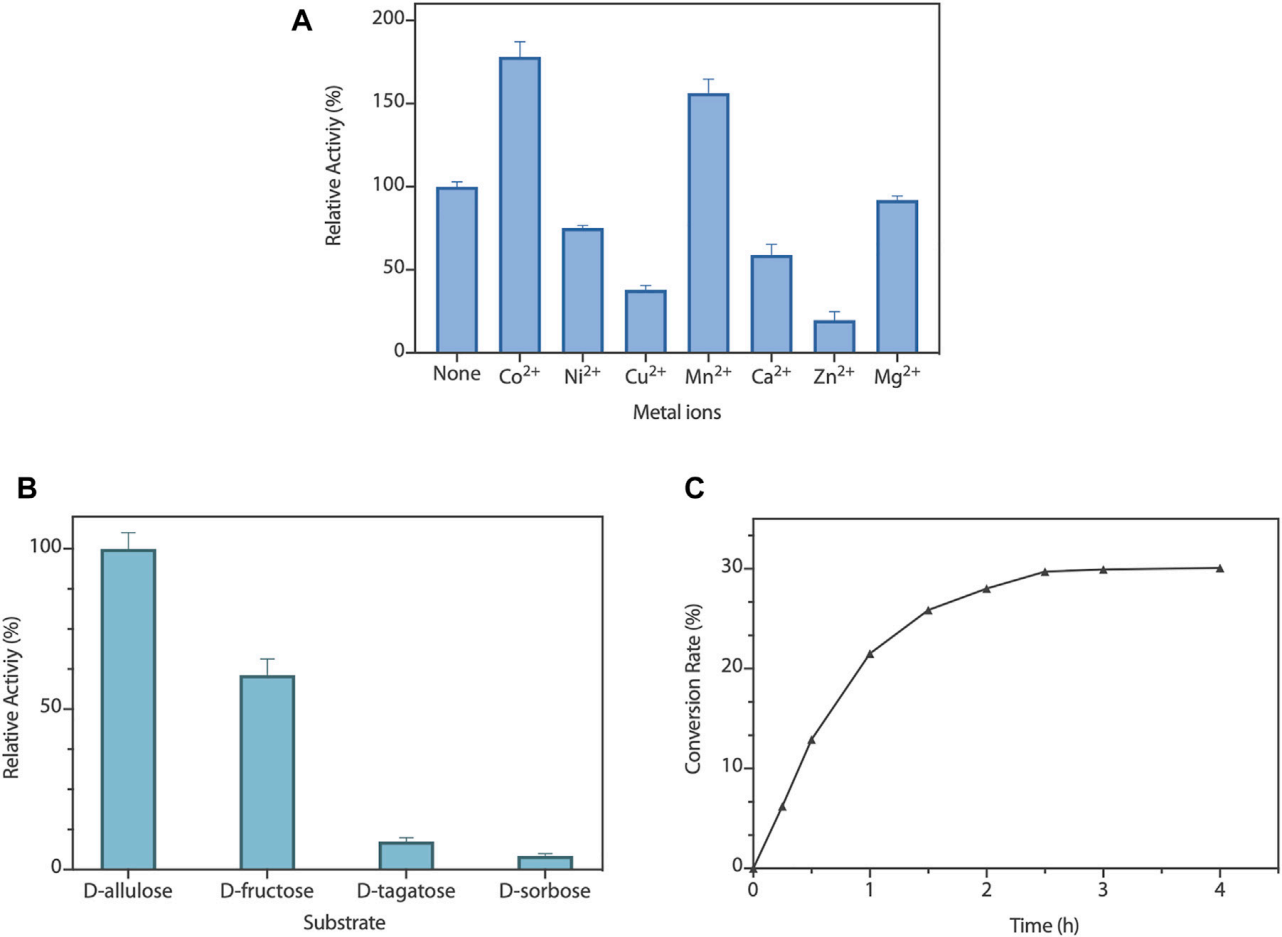
**(A)** Effect of metal ions on RpDAE activity. **(B)** Substrate specificity of RpDAE. **(C)** Time course of bioconversion from D-fructose to D-allulose by RpDAE.

To capture the effect of metal ions on RpDAE activity, diverse divalent metal ions were added into the reaction system at the final concentration of 1 mM. As illustrated in Figure 4A, RpDAE displayed activity in the absence of metal ions and enhanced in the presence of Co^2+^ and Mn^2+^, by 1.78- and 1.56-fold, respectively. In contrast, the addition of Cu^2+^, Ca^2+^, Zn^2+^, and Ni^2+^ inhibited enzyme activity, and the inhibitory effect of Zn^2+^ was the most significant, over 80%. Moreover, Mg^2+^ had little effect on the relative activity of RpDAE. Monosaccharide epimerase employing deprotonation/reprotonation mechanism often required metal ions as co-factor to participate in the catalysis (Van Overtveldt et al., 2015), and the optimum co-factor for RpDAE was determined to be Co^2+^.

To explore the substrate specificity of RpDAE, four kinds of ketoses were used, containing D-allulose, D-fructose, D-tagatose, and D-sorbose. RpDAE displayed the highest activity in the presence of D-allulose, which was 40% higher in relation to D-fructose (Figure 4B). On the contrary, RpDAE displayed low epimerization activity toward D-tagatose and D-sorbose.

### Bioconversion of D-Allulose

The large-scale bioconversion of D-allulose was performed with D-fructose (500 g/L), 1 mM Co^2+^, and 50 mM KH_2_PO_4_/Na_2_HPO_4_ (pH 7.5), along with 0.2 g/L RpDAE at 50°C. The reaction rate for D-allulose production was 107.5 g/h for the first hour. Finally, the equilibrium ratio of D-allulose and D-fructose was measured to be 29.9:70.1, and D-allulose (approximately 149.5 g/L) were obtained from D-fructose (500 g/L) without byproducts after 150 min (Figure 4C). At present, many multinational enterprises are researching and developing the biological production of D-allulose. In 2021, the European Food Safety Authority certified the safety of the two kinds of food enzyme DAEs, which were produced by genetically modified *E. coli* K-12 W3110 (pWKLP) strain (Matsutani Chemical Industry Co., Ltd.) (Lambré et al., 2021a) and *Corynebacterium Glutamicum* FIS002 strain (CJ-Tereos Sweeteners Europe SAS) (Lambré et al., 2021b), respectively. The efficiency of biocatalysts is crucial in industrial production. Generally, the conversion rates of different DAEs reported in the literature were between 22% and 32.9% (Jia et al., 2021), and the highest conversion yield was achieved by *A. tumefaciens* ATCC33970 DAE (Kim et al., 2006a). Compared to reported DAEs, the catalytic performance of RpDAE at the substrate scale of 500 g/L was at middle-upper levels, revealing as a potential candidate in the biological production of D-allulose.

### Effect of Temperature and pH on RpDAE

**FIGURE 5:**
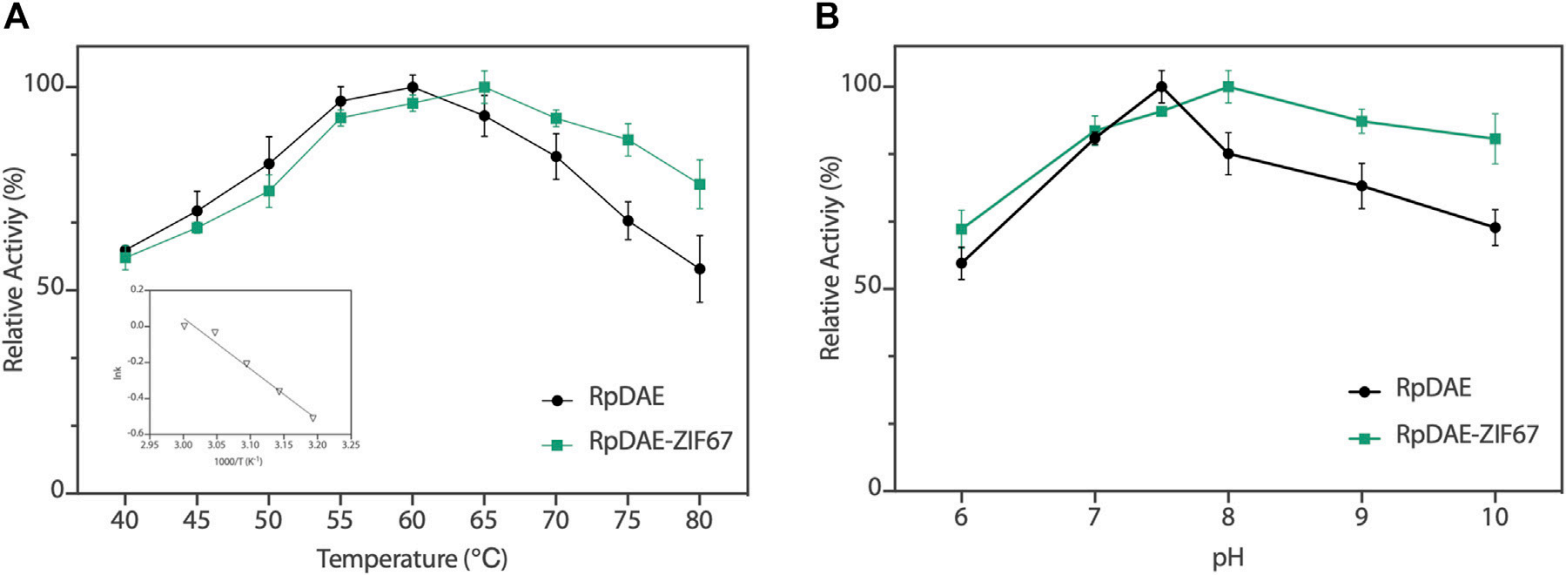
**(A)** Temperature−activity profile of RpDAE and RpDAE-ZIF67. **(B)** pH−activity profile of RpDAE and RpDAE-ZIF67.

The enzyme activity of RpDAE was dependent on temperature and pH conditions. The influence of the temperatures on RpDAE activity was depicted in Figure 5A, and the activity was assayed with a temperature range of 40°C–80°C at pH 7.5. RpDAE displayed more than 82.9% relative activity at the temperature range between 55°C and 70°C. The maximum enzymatic activity of RpDAE was recorded at 60°C, which was higher than optimum temperature of *C. cellulolyticum* DAE (Mu et al., 2011). To further determine the activation energy (*E*_*a*_) of epimerization reaction at pH 7.5, activities measured at 40°C–60°C were plotted as ln (relative activity) versus 1000/T, and Arrhenius equation [ln*k* = (−*E*_*a*_/*RT*) + ln*A*] was used to calculate the *E*_*a*_ of 23.51 kJ/mol (illustration of Figure 5A).

The effect of pH on RpDAE was examined at 60°C over a pH range from 6.0 to 10.0, and the optimal pH occurred at a weak level of alkaline, which was pH 7.5 (Figure 5B). Most characterized DAEs showed optimum epimerization activity at pH 7.0 to 9.0, and the exception was *Dorea* sp. DAE with an optimal pH at 6.0 (Zhang et al., 2015). RpDAE showed more than 87% of relative activity at pH 7.0, demonstrating application potentials in D-allulose production, because neutral conditions help to reduce browning of monosaccharide, thereby reducing yield loss.

### Preparation and Characterization of RpDAE-ZIF67

**FIGURE 6:**
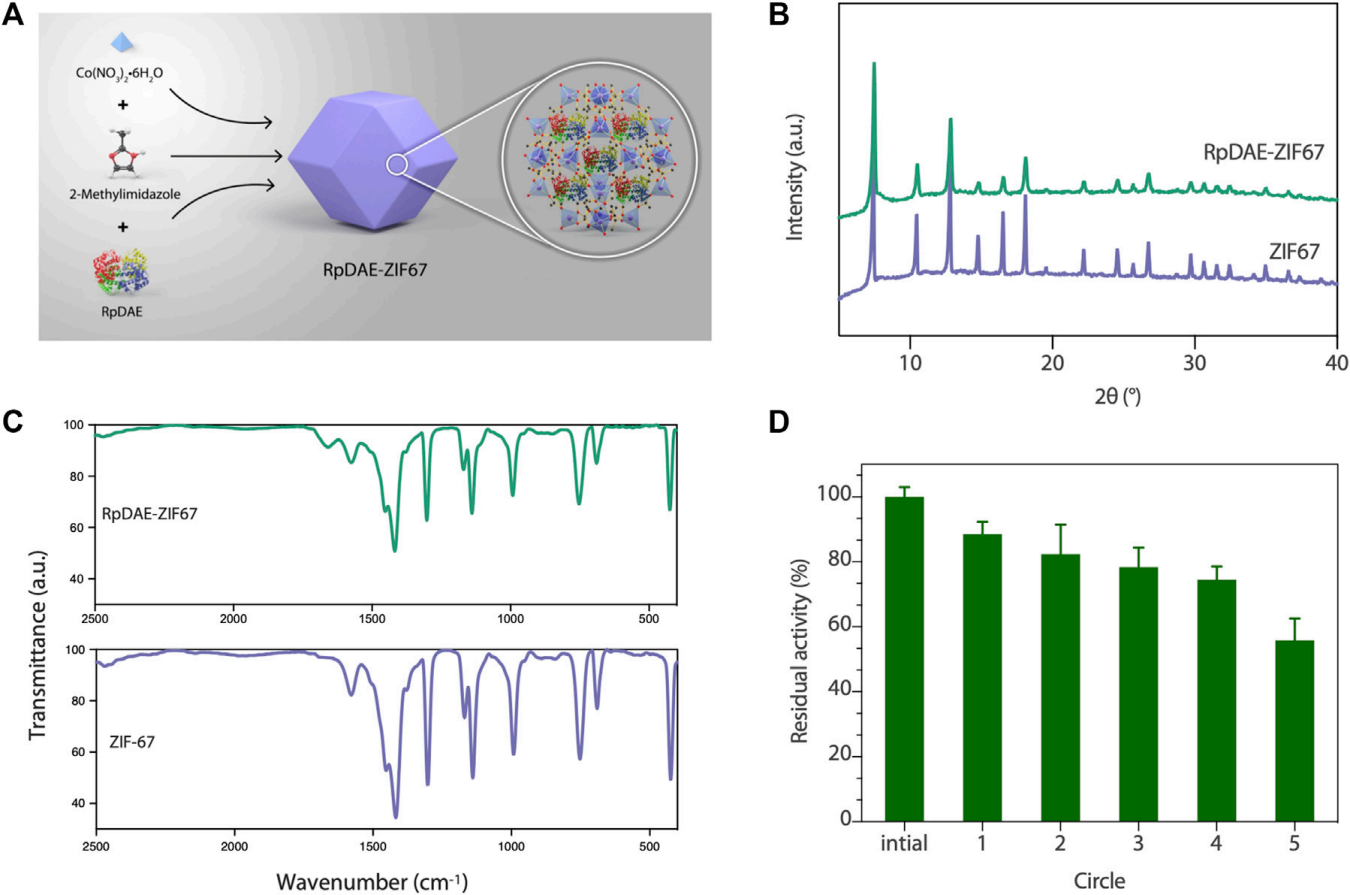
**(A)** Scheme of synthesis of RpDAE-ZIF67 bio-composites. **(B)** PXRD patterns of RpDAE-ZIF67 and ZIF67. **(C)** FT-IR spectra of RpDAE-ZIF67 and ZIF67. **(D)** Cycle stability of RpDAE-ZIF67.

Immobilized enzymes are widely utilized in the food industry. On the one hand, immobilized enzymes are heterogeneous catalysts so that can be simply separated from the reaction medium and obtain a pure product without contamination. On the other hand, they can be applied multiple times to the production process, thus reducing costs (Yushkova et al., 2019). Benefiting from high biocompatibility and mild synthesis conditions, ZIFs are the most widely used *in situ* immobilization matrices (Lian et al., 2017). RpDAE-ZIF67 was synthesized through crystallization of ZIF67 around RpDAE in aqueous solution (Figure 6A), which is a straightforward, rapid, and cost-effective process (Patil and Yadav, 2018). To remove loosely attached RpDAE on the surface, the obtained RpDAE-ZIF67 particles were washed three times with deionized water. The synthesized ZIF67 and RpDAE-ZIF67 were characterized by PXRD. As shown in Figure 6B, the conspicuous reflections of synthesized ZIF67 at 2θ = 7.5°, 10.5°, 12.9°, 14.8°, 16.6°, 18.1°, 22.2°, 24.6°, 25.7°, 26.8°, 29.7°, 30.7°, 31.6°, and 32.5° were associated with (0 1 1), (0 0 2), (1 1 2), (0 2 2), (0 1 3), (2 2 2), (1 1 4), (2 3 3), (2 2 4), (1 3 4), (0 4 4), (3 3 4), (2 4 4), and (2 3 5), respectively, of the simulated ZIF67 single-crystal planes (Banerjee et al., 2008) (Guo et al., 2016). Similar diffraction patterns were also observed in RpDAE-ZIF67, which indicated that the crystal structure of ZIF67 remained unaffected after the enzyme being incorporated. Figure 6C showed the FT-IR spectra of RpDAE and RpDAE-ZIF67. The vibrational bands of bare ZIF67 in the range of 600–1,500 cm^−1^ correspond to the characteristic stretch and bending modes of imidazole rings. Furthermore, the band at 1,574 cm^−1^ can be attributed to the stretching mode of C=N in 2-methylimidazole. The above bands were all well represented in the spectrum of RpDAE-ZIF67. In addition, RpDAE-ZIF67 had a new absorption band at 1,658 cm^−1^ compared to bare ZIF67, corresponding to the stretching vibration mode of C=O in the amide I bond, confirming the existence of DAE within ZIF67.

The relative activities of encapsulated DAE were evaluated, and Figure 5A demonstrated nearly identical activities of RpDAE and RpDAE-ZIF67 under 60°C. However, at higher temperatures (over 65°C), the encapsulated DAE displayed higher relative activity than free DAE. At 80°C, the free DAE showed only 55.3% of relative activity, whereas the encapsulated DAE retained 76.1% of relative activity. These results demonstrated that encapsulating enzymes in MOFs prevented conformational transitions at high temperatures and improved the thermostability. As shown in Figure 5B, encapsulated DAE reached maximum activity at pH 8.0 and showed the higher tolerance in alkaline compared with free DAE. Another purpose of immobilization is to make the biocatalyst easy to recover and reuse, which is a key factor for economic viability. Hence, the reusability of RpDAE was examined in consecutive epimerization reactions. Accordingly, the encapsulated DAE was separated from the reaction system by centrifugation. As shown in Figure 6D, the encapsulated DAE was successfully cycled for five times, and the residual activity was determined to be 56%. Wang et al. immobilized laccase within ZIF67 by the one-pot synthesis strategy, which maintained 59% residual enzyme activity after five reaction cycles (Wang et al., 2020). The gradual loss of activity during recycling may cause by mechanical damage to the enzyme. The reusability can be attributed to the small window size of the cages, physically preventing the leaching of the enzymes from ZIF67.

## Conclusion

In the present study, a novel RpDAE was identified, overexpressed in *E. coli*, purified, and characterized. RpDAE activity was not dependent on the presence of metal ions and can be enhanced by cobalt ions. The application potential was evaluated by large-scale bioconversion from D-fructose to D-allulose. Further, we pioneered the immobilization of DAE within ZIF67 by *in situ* encapsulation strategy. Because of the shielding effect, the immobilized DAE exhibited reusability. These results provide an important reference for further research on the biological industrial production of D-allulose.

## Data Availability

The datasets presented in this study can be found in online repositories. The names of the repository/repositories and accession number(s) can be found in the article/Supplementary Material.

## References

[B2] Ayers B. J. Hollinshead J. Saville A. W. Nakagawa S. Adachi I. Kato A. (2014). Iteamine, the First Alkaloid Isolated from Itea Virginica L. Inflorescence. Phytochemistry 100, 126–131. 10.1016/j.phytochem.2014.01.012 24534106

[B3] Banerjee R. Phan A. Wang B. Knobler C. Furukawa H. O'Keeffe M. (2008). High-Throughput Synthesis of Zeolitic Imidazolate Frameworks and Application to CO 2 Capture. Science 319 (5865), 939–943. 10.1126/science.1152516 18276887

[B4] Chan H.-C. Zhu Y. Hu Y. Ko T.-P. Huang C.-H. Ren F. (2012). Crystal Structures of D-Psicose 3-epimerase from Clostridium Cellulolyticum H10 and its Complex with Ketohexose Sugars. Protein Cell 3 (2), 123–131. 10.1007/s13238-012-2026-5 22426981 PMC4875416

[B5] Chen D. Chen J. Liu X. Guang C. Zhang W. Mu W. (2021). Biochemical Identification of a Hyperthermostable L-Ribulose 3-epimerase from Labedella Endophytica and its Application for D-Allulose Bioconversion. Int. J. Biol. Macromolecules 189, 214–222. 10.1016/j.ijbiomac.2021.08.131 34428486

[B6] Chung M.-Y. Oh D.-K. Lee K. W. (2012). Hypoglycemic Health Benefits of D-Psicose. J. Agric. Food Chem. 60 (4), 863–869. 10.1021/jf204050w 22224918

[B7] Cree G. M. Perlin A. S. (1968). O-isopropylidene Derivatives of D-Allulose (D-Psicose) and D-Erythro-Hexopyranos-2,3-Diulose. Can. J. Biochem. 46 (8), 765–770. 10.1139/o68-117 4299740

[B8] EFSA Panel on Food Contact Materials, Enzymes and Processing Aids (CEP) Lambré C. Barat Baviera J. M. Bolognesi C. Cocconcelli P. S. Crebelli R. Gott D. M. (2021a). Safety Evaluation of the Food Enzyme D‐psicose 3‐epimerase from the Genetically Modified *Escherichia coli* Strain K‐12 W3110 (pWKLP). EFS 19 (4), 6870. 10.2903/j.efsa.2021.6870 PMC8085949 33968253

[B9] EFSA Panel on Food Contact Materials, Enzymes and Processing Aids (CEP) Lambré C. Barat Baviera J. M. Bolognesi C. Cocconcelli P. S. Crebelli R. Gott D. M. (2021b). Safety Evaluation of the Food Enzyme D‐psicose 3‐epimerase from the Genetically Modified Corynebacterium Glutamicum Strain FIS002. EFS 19 (10), 6870. 10.2903/j.efsa.2021.6870 PMC8521546 34703502

[B10] Emmadi M. Kulkarni S. S. (2014). Recent Advances in Synthesis of Bacterial Rare Sugar Building Blocks and Their Applications. Nat. Prod. Rep. 31 (7), 870–879. 10.1039/c4np00003j 24700208

[B11] Fang Z. Li T. Wang Q. Zhang X. Peng H. Fang W. (2011). A Bacterial Laccase from marine Microbial Metagenome Exhibiting Chloride Tolerance and Dye Decolorization Ability. Appl. Microbiol. Biotechnol. 89 (4), 1103–1110. 10.1007/s00253-010-2934-3 20963410

[B12] Gross A. F. Sherman E. Vajo J. J. (2012). Aqueous Room Temperature Synthesis of Cobalt and Zinc Sodalite Zeolitic Imidizolate Frameworks. Dalton Trans. 41 (18), 5458. 10.1039/c2dt30174a 22406684

[B13] Guo X. Xing T. Lou Y. Chen J. (2016). Controlling ZIF-67 Crystals Formation through Various Cobalt Sources in Aqueous Solution. J. Solid State. Chem. 235, 107–112. 10.1016/j.jssc.2015.12.021

[B14] Han Y. Guo W. Su B. Guo Y. Wang J. Chu B. (2018). High-level Expression of Soluble Recombinant Proteins in *Escherichia coli* Using an HE-Maltotriose-Binding Protein Fusion Tag. Protein Expr. Purif. 142, 25–31. 10.1016/j.pep.2017.09.013 28963004

[B15] Hofström C. Orlova A. Altai M. Wångsell F. Gräslund T. Tolmachev V. (2011). Use of a HEHEHE Purification Tag Instead of a Hexahistidine Tag Improves Biodistribution of Affibody Molecules Site-Specifically Labeled with ^99m^ Tc, ^111^ in, and ^125^ I. J. Med. Chem. 54 (11), 3817–3826. 21524142 10.1021/jm200065e

[B16] Homaei A. A. Sariri R. Vianello F. Stevanato R. (2013). Enzyme Immobilization: an Update. J. Chem. Biol. 6 (4), 185–205. 10.1007/s12154-013-0102-9 24432134 PMC3787205

[B17] Jia D.-X. Sun C.-Y. Jin Y.-T. Liu Z.-Q. Zheng Y.-G. Li M. (2021). Properties of D-Allulose 3-epimerase Mined from Novibacillus Thermophilus and its Application to Synthesis of D-Allulose. Enzyme Microb. Technology 148, 109816. 10.1016/j.enzmictec.2021.109816 34116747

[B18] Johnson R. J. Segal M. S. Sautin Y. Nakagawa T. Feig D. I. Kang D.-H. (2007). Potential Role of Sugar (Fructose) in the Epidemic of Hypertension, Obesity and the Metabolic Syndrome, Diabetes, Kidney Disease, and Cardiovascular Disease1Ϫ3. Am. J. Clin. Nutr. 86 (4), 899–906. 10.1093/ajcn/86.4.899 17921363

[B20] Kim H.-J. Hyun E.-K. Kim Y.-S. Lee Y.-J. Oh D.-K. (2006). Characterization of an Agrobacterium Tumefaciens D -Psicose 3-Epimerase that Converts D -Fructose to D -Psicose. Appl. Environ. Microbiol. 72 (2), 981–985. 10.1128/aem.72.2.981-985.2006 16461638 PMC1392948

[B21] Kim K. Kim H.-J. Oh D.-K. Cha S.-S. Rhee S. (2006). Crystal Structure of D-Psicose 3-epimerase from Agrobacterium Tumefaciens and its Complex with True Substrate D-Fructose: A Pivotal Role of Metal in Catalysis, an Active Site for the Non-phosphorylated Substrate, and its Conformational Changes. J. Mol. Biol. 361 (5), 920–931. 10.1016/j.jmb.2006.06.069 16876192

[B22] Lian X. Fang Y. Joseph E. Wang Q. Li J. Banerjee S. (2017). Enzyme-MOF (Metal-organic Framework) Composites. Chem. Soc. Rev. 46 (11), 3386–3401. 10.1039/c7cs00058h 28451673

[B23] Liang K. Ricco R. Doherty C. M. Styles M. J. Bell S. Kirby N. (2015). Biomimetic Mineralization of Metal-Organic Frameworks as Protective Coatings for Biomacromolecules. Nat. Commun. 6 (1), 7240. 10.1038/ncomms8240 26041070 PMC4468859

[B24] Lyu F. Zhang Y. Zare R. N. Ge J. Liu Z. (2014). One-Pot Synthesis of Protein-Embedded Metal-Organic Frameworks with Enhanced Biological Activities. Nano Lett. 14 (10), 5761–5765. 10.1021/nl5026419 25211437

[B19] Miller B. S. Swain T. (1960). Chromatographic Analyses of the Free Amino-Acids, Organic Acids and Sugars in Wheat Plant Extracts. J. Sci. Food Agric. 11 (6), 344–348.

[B26] Mu W. Chu F. Xing Q. Yu S. Zhou L. Jiang B. (2011). Cloning, Expression, and Characterization of a D-Psicose 3-Epimerase from Clostridium Cellulolyticum H10. J. Agric. Food Chem. 59 (14), 7785–7792. 10.1021/jf201356q 21663329

[B27] Mu W. Yu L. Zhang W. Zhang T. Jiang B. (2015). Isomerases for Biotransformation of D-Hexoses. Appl. Microbiol. Biotechnol. 99 (16), 6571–6584. 10.1007/s00253-015-6788-6 26150247

[B28] Oshima H. Kimura I. Zumori K. (2006). Psicose Contents in Various Food Products and its Origin. Food Sci. Technol. Res. 12 (2), 137–143.

[B29] Patil P. D. Yadav G. D. (2018). Rapid *In Situ* Encapsulation of Laccase into Metal-Organic Framework Support (ZIF-8) under Biocompatible Conditions. ChemistrySelect 3 (17), 4669–4675. 10.1002/slct.201702852

[B30] Rafiei S. Tangestaninejad S. Horcajada P. Moghadam M. Mirkhani V. Mohammadpoor-Baltork I. (2018). Efficient Biodiesel Production Using a lipase@ZIF-67 Nanobioreactor. Chem. Eng. J. 334, 1233–1241. 10.1016/j.cej.2017.10.094

[B1] Trends in Adult Body-Mass (2016) index in 200 Countries from 1975 to 2014: a Pooled Analysis of 1698 Population-Based Measurement Studies with 19·2 Million Participants. Lancet, 387 (10026), 1377–1396. 10.1016/S0140-6736(16)30054-X 27115820 PMC7615134

[B31] Van Overtveldt S. Verhaeghe T. Joosten H.-J. van den Bergh T. Beerens K. Desmet T. (2015). A Structural Classification of Carbohydrate Epimerases: From Mechanistic Insights to Practical Applications. Biotechnol. Adv. 33 (8), 1814–1828. 10.1016/j.biotechadv.2015.10.010 26505535

[B32] Vorapreeda T. Thammarongtham C. Cheevadhanarak S. Laoteng K. (2015). Genome Mining of Fungal Lipid-Degrading Enzymes for Industrial Applications. Microbiology 161 (8), 1613–1626. 10.1099/mic.0.000127 26271808

[B33] Wang Z. Ren D. Yu H. Jiang S. Zhang S. Zhang X. (2020). Study on Improving the Stability of Adsorption-Encapsulation Immobilized Laccase@ZIF-67. Biotechnol. Rep. 28, e00553. 10.1016/j.btre.2020.e00553 PMC7674278 33240797

[B34] Wyss F. Coca A. Lopez-Jaramillo P. Ponte-Negretti C. Wyss F. S. Restrepo G. (2020). Position Statement of the Interamerican Society of Cardiology (IASC) on the Current Guidelines for the Prevention, Diagnosis and Treatment of Arterial Hypertension 2017-2020. Int. J. Cardiol. Hypertens. 6, 100041. 10.1016/j.ijchy.2020.100041 33447767 PMC7803017

[B35] Xia Y. Cheng Q. Mu W. Hu X. Sun Z. Qiu Y. (2021). Research Advances of D-Allulose: An Overview of Physiological Functions, Enzymatic Biotransformation Technologies, and Production Processes. Foods 10 (9), 2186. 10.3390/foods10092186 34574296 PMC8467252

[B36] Yoshida H. Yoshihara A. Gullapalli P. K. Ohtani K. Akimitsu K. Izumori K. (2018). X-ray Structure of Arthrobacter Globiformis M30 Ketose 3-epimerase for the Production of D-Allulose from D-Fructose. Acta Cryst. Sect F 74 (10), 669–676. 10.1107/s2053230x18011706 PMC6168773 30279320

[B37] Yushkova E. D. Nazarova E. A. Matyuhina A. V. Noskova A. O. Shavronskaya D. O. Vinogradov V. V. (2019). Application of Immobilized Enzymes in Food Industry. J. Agric. Food Chem. 67 (42), 11553–11567. 10.1021/acs.jafc.9b04385 31553885

[B38] Zepeda V. Dassa B. Borovok I. Lamed R. Bayer E. A. Cate J. H. (2013). Draft Genome Sequence of the Cellulolytic Bacterium Clostridium Papyrosolvens C7 (ATCC 700395). Genome Announc 1 (5), e00698–13. 10.1128/genomeA.00698-13 24029755 PMC3772139

[B39] Zhang W. Fang D. Zhang T. Zhou L. Jiang B. Mu W. (2013). Characterization of a Metal-dependent D-Psicose 3-Epimerase from a Novel Strain, Desmospora Sp. 8437. J. Agric. Food Chem. 61 (47), 11468–11476. 10.1021/jf4035817 24199681

[B40] Zhang W. Li H. Zhang T. Jiang B. Zhou L. Mu W. (2015). Characterization of a D-Psicose 3-epimerase from Dorea Sp. CAG317 with an Acidic pH Optimum and a High Specific Activity. J. Mol. Catal. B: Enzymatic 120, 68–74. 10.1016/j.molcatb.2015.05.018

[B41] Zou Z.-Z. Yu H.-L. Li C.-X. Zhou X.-W. Hayashi C. Sun J. (2012). A New Thermostable β-glucosidase Mined from Dictyoglomus Thermophilum: Properties and Performance in Octyl Glucoside Synthesis at High Temperatures. Bioresour. Technology 118, 425–430. 10.1016/j.biortech.2012.04.040 22705966

